# Unexpected effects of pandemic-related changes on mental health: Evidence from a nationwide survey in South Korea

**DOI:** 10.1371/journal.pone.0317493

**Published:** 2025-01-13

**Authors:** Won-Hyoung Kim, Jonghun Kim, Jiyun Oh, Seolim Lee, Jihwan Chang, Younhee Kim

**Affiliations:** 1 Department of Psychiatry and Mental Health, College of Medicine, Inha University, Incheon, South Korea; 2 College of Medicine, Inha University, Incheon, South Korea; 3 Department of Social and Preventive Medicine, College of Medicine, Inha University, Incheon, South Korea; Universita Cattolica del Sacro Cuore Sede di Roma, ITALY

## Abstract

**Background:**

Substantial evidence indicates that the COVID-19 pandemic has adversely affected population mental health globally. However, most studies assumed a linear relationship where only negative pandemic-induced changes led to worse mental health outcomes, overlooking the complex relationship between COVID-19-related changes and mental health. This study examined how various types and magnitudes of pandemic-related changes relate to depression and suicidal thoughts in a large, nationwide adult population sample.

**Methods:**

We analyzed data from the 2021 Korean Community Health Survey, a cross-sectional survey of 229,213 adults. The study examined the association between mental health outcomes and three types of pandemic-related changes: daily life impact (scored 0–100), economic activities (employment and income changes), and health behaviors (physical activity, instant food consumption, alcohol consumption, and smoking). Complex sample multiple logistic regression analysis was used to assess these associations, adjusting for sociodemographic factors.

**Results:**

The relationship between pandemic-related changes and mental health showed non-linear patterns. Compared to those reporting moderate changes, individuals reporting either no change (depression: aOR 1.253, 95% CI 1.135–1.384; suicidal thoughts: aOR 1.355, 95% CI 1.236–1.486) or complete disruption (depression: aOR 1.895, 95% CI 1.667–2.155; suicidal thoughts: aOR 1.788, 95% CI 1.558–2.052) in daily life showed higher risks of poor mental health. Unexpectedly, positive changes such as improved working conditions (suicidal thoughts: aOR 1.419, 95% CI 1.200–1.677) and increased income (depression: aOR 1.304, 95% CI 1.139–1.493; suicidal thoughts: aOR 1.244, 95% CI 1.079–1.435) were also associated with adverse mental health outcomes.

**Conclusions:**

This study reveals that both minimal and substantial changes in daily life, as well as both positive and negative changes in economic conditions and health behaviors, were associated with poor mental health outcomes during the pandemic. These findings suggest the need for comprehensive mental health interventions that consider various types and magnitudes of life changes during crisis situations.

## Introduction

Mental health is an integral part of our general health and well-being and a fundamental human right [1]. The World Health Organization emphasizes that good mental health enables individuals to better connect, function, cope, and thrive in their daily lives.

The COVID-19 pandemic has introduced unprecedented psychological stressors affecting mental health globally. These stressors are categorized into four primary dimensions [2, 3]. First, direct health-related stressors emerged from COVID-19 exposure and infection concerns. These stressors manifested through multiple pathways: fear or anxiety regarding potential infection, experiential trauma from witnessing disease progression in oneself or close contacts, and psychological distress from mortality-related encounters. Multiple studies have provided empirical evidence that COVID-19-related fear and anxiety are significantly associated with adverse mental health outcomes [4–6]. Second, the implementation of public health and social measures, including lockdowns, social distancing, self-isolation, remote work, and school closures, has created another significant source of stress. Research indicates that these measures have led to widespread feelings of isolation and loneliness, resulting in deteriorated mental health [7–11]. These measures particularly affected the mental health of parents caring for children during school closures, as they experienced increased parenting burden and family conflicts, leading to deterioration in mental health [12, 13]. Third, the economic crisis triggered by the pandemic has caused widespread unemployment and financial insecurity, leading to economic distress. Empirical studies have identified job loss and income reduction as significant risk factors for mental health deterioration [14, 15]. Fourth, the pervasive spread of misinformation and uncertainty surrounding the pandemic has generated substantial psychological burden. Several studies have provided evidence linking pandemic-related misinformation and uncertainty to increased depression and anxiety levels [16–18].

These multiple stressors present unprecedented challenges to mental health resilience [19–26]. Even individuals with previously effective stress coping strategies struggled to maintain psychological stability during the COVID-19 pandemic, while those with pre-existing mild depression or stress were likely to experience symptom exacerbation. Moreover, individuals with pre-existing mental health conditions faced potentially severe deterioration due to multiple stressors [27–29]. This vulnerability was further exacerbated by reduced access to mental health services during the pandemic [28, 30].

Meanwhile, studies examining suicidal behaviors during the COVID-19 pandemic have yielded mixed results. While some research has reported increased suicidal behaviors [11, 21, 31–33], others have observed no significant increase or even decreased rates [19, 31–35]. This variation may be partially explained by protective factors that emerge during disasters. During disasters, such as infectious diseases or terrorist attacks, enhanced social cohesion within communities may contribute to an initial decline in suicidal behaviors [36, 37]. Furthermore, the increased time spent at home during the pandemic could result in emotional and physical support from family members, potentially positively affecting mental health of individuals. However, despite these potential protective mechanisms, previous studies consistently suggest the possibility of accumulating risk factors for depression and suicidal behaviors as the pandemic persists [38]. Additionally, even if the average level of mental health in the general population has not significantly changed during the pandemic, there remains a need to study groups experiencing changes in various life domains [39, 40] and socially vulnerable populations [41].

In response to the mental health challenges posed by the COVID-19 pandemic, South Korean health authorities implemented several Mental Health and Psychological Interventions to monitor and support the psychological well-being of the population [42–44]. One key effort was the initiation of the COVID-19 National Mental Health Survey in March 2020. This quarterly survey aimed to assess the mental health status of the general population, including levels of depression, anxiety, stress, and suicide risk, allowing continuous monitoring to track changes in mental health indicators throughout the pandemic and adjust interventions accordingly. Furthermore, the Korean government established an integrated psychological support system early in the pandemic, organizing the National Center for Disaster Trauma to coordinate a network of national and local mental health welfare centers for providing preemptive interventions to reduce mental distress. This system offered 24-hour counseling services, mental health assessments, and referrals to specialized medical institutions for high-risk individuals. The Korean Neuropsychiatric Association also published mental health guidelines targeting five population domains: the general population, parents of young children, quarantined individuals, medical professionals treating COVID-19 patients, and other medical practitioners. These guidelines, distributed by the Ministry of Health and Welfare, emphasized the importance of accepting anxiety as a normal emotion, obtaining reliable information, and paying attention to vulnerable populations. Despite these efforts, numerous studies have reported that COVID-19 has had multiple negative impacts on the mental health of both infected and non-infected individuals in South Korea [14, 45–48].

While many studies have reported that COVID-19-related changes negatively affect mental health, most studies have only adopted a linear relationship where pandemic-induced negative changes necessarily lead to worse mental health outcomes [38, 49–53]. These current studies have overlooked the complex and multifaceted aspects of the relationship between COVID-19-related changes and mental health: the mental health status of individuals reporting no impact from the pandemic, the potential psychological burden associated with positive economic changes (such as increased income or improved working conditions), and the relationship between both positive and negative changes in health behaviors and mental health. Additionally, most studies have examined specific populations [5, 29, 54–56] or relied on convenience sampling, limiting their generalizability to the general population.

This study aims to address these gaps by comprehensively examining how various types and degrees of pandemic-related changes relate to depression and suicidal thoughts in a large, representative adult population sample. These changes include minimal changes in daily life due to COVID-19, extreme disruptions, and both positive and negative changes in economic conditions and health behaviors. Understanding these complex relationships will be crucial for developing more targeted and effective mental health interventions during public health crises.

## Methods

### Data source

Data were obtained from the 2021 Korean Community Health Survey (KCHS), an annual nationwide survey administered by the Korea Disease Control and Prevention Agency (KDCA). The KCHS generates regional-level health statistics to facilitate the implementation of community health programs and is classified as official national statistics in South Korea. The target population consisted of individuals aged 19 years and older residing within the jurisdictions of all 255 community health centers across South Korea. While maintaining consistency with the fundamental sampling design described in previous studies [57], several significant methodological improvements have been implemented in recent years. Since 2018, the survey has incorporated direct physical measurements for height and weight, replacing self-reported data, and in 2019, blood pressure measurements using standardized sphygmomanometers were introduced. In particular, the 2021 KCHS expanded to include a comprehensive pandemic response module designed to assess COVID-19’s impact across multiple domains, including questions about adherence to personal hygiene and preventive measures (e.g., handwashing, mask-wearing, and physical distancing), changes in employment status and household income, COVID-19-related concerns (e.g., fear of infection, worry about social stigma, concerns about economic damage), COVID-19 vaccination-related questions, and modifications in health behaviors including physical activity, alcohol consumption, and smoking [58]. The 2021 survey ultimately comprised 163 questions across 18 domains, incorporating both individual and household-level assessments.

This study was approved by the Institutional Review Board of Inha University Hospital (IRB No. INHAUH 2023-09-012). Written informed consent was not required for this study as it analyzed publicly available secondary data from the KCHS, which contains no personal identifiers.

### Variables

Participants were asked the following question regarding depressive experience: “Have you felt sad or desperate to the extent that it interfered with your daily life for more than two weeks in the past year?” The responses were limited to “Yes” or “No,” with “Yes” indicating that the individual has had a depressive experience in the past year. Suicidal thought was assessed using the question: “Have you thought about wanting to die in the past year?” Again, the response options were “Yes” or “No”. Given that the survey was conducted from August 16 to October 31, 2021, the period “past year” encompasses the duration of the COVID-19 pandemic.

The main explanatory variables were changes in daily life, economic activities, and health behaviors due to the COVID-19 pandemic. First, participants rated the overall impact of the pandemic on their daily life on a scale from 0 to 100, where initially 0 indicated a complete standstill and 100 indicated unchanged daily life. Responses were recorded at 10-point intervals. To facilitate interpretation, these scores were inverted so that 0 represented unchanged daily life and 100 indicated complete disruption. Participants who rated the impact as 50 points (indicating moderate changes) served as the reference group.

Second, changes in economic activities were assessed in two domains: employment and household income. Employment changes were classified as “unchanged” (reference category), “lost a job,” “work environment worsened,” “work environment improved,” or “did not engage in economic activity even before the pandemic.” Household income changes were classified as “decreased,” “unchanged” (reference category), or “increased.”

Third, changes in health-related behaviors included physical activity, instant food consumption, alcohol consumption, and smoking. For each behavior, responses were categorized as “increased,” “unchanged” (reference category), “decreased,” or “not applicable.” The “not applicable” category referred to individuals who did not engage in these activities even before COVID-19.

The confounding variables were age, sex (male and female), education level (less than middle school, high school, and college or above), monthly income (below and above 3 million Korean won), marital status (married, widowed, divorced, and single), self-rated health (very good, good, fair, poor, and very poor), household composition (living alone and living with others), and residence (urban and rural areas).

### Statistical analysis

Statistical analyses were conducted using weights, stratification variables, and clustering variables to reflect the complex sample design. Descriptive analysis presented the sociodemographic characteristics of participants and patterns of pandemic-related changes in daily life, employment, household income, and health behaviors as frequencies and weighted percentages. The Rao-Scott chi-square test compared the prevalence of depression and suicidal thought across different characteristics.

Complex-sample multiple logistic regression analyses examined the association between various life changes during the pandemic and the prevalence of depression and suicidal thought after adjusting for control variables. The results were reported as adjusted odds ratios (aORs) with 95% confidence intervals (CIs).

All data processing and statistical analyses were performed with SAS version 9.4 (SAS Institute, Cary, NC, USA), using PROC SURVEY procedures to account for the complex stratified cluster sampling design.

## Results

### Participant characteristics by mental health status

Sociodemographic characteristics based on depression and suicidal thoughts are presented in Table 1. Of the 229,213 participants, 7.17% reported experiencing depression and 6.52% had suicidal thoughts in the past year. The prevalence differed by sex, with 5.29% of males and 9.03% of females reporting depression, and 4.82% of males and 8.19% of females reporting suicidal thoughts. Depression and suicidal thoughts increased with age, particularly among those aged 70 years and older (depression: 8.72%; suicidal thoughts: 10.07%). Higher rates of depression and suicidal thoughts were found among participants with lower education and income levels, and those who were widowed or divorced. Poor self-rated health and living alone were also associated with higher rates of both outcomes.

**Table 1:** Sociodemographic characteristics by depression and suicidal thoughts.

	Total	Depression		Suicidal thoughts	
	n (weighted %)	n (weighted %)	P value	n (weighted %)	P value
Total	229,213 (100.00%)	16,332 (7.17%)		16,070 (6.52%)	
Sex			<0.001		<0.001
Male	104,489 (49.58%)	5,382 (5.29%)		5,340 (4.82%)	
Female	124,724 (50.42%)	10,950 (9.03%)		10,730 (8.19%)	
Age group			<0.001		<0.001
19–29	24,695 (16.63%)	1,494 (6.30%)		1,346 (5.47%)	
30–39	25,731 (15.51%)	1,719 (6.31%)		1,511 (5.58%)	
40–49	35,531 (18.81%)	2,323 (6.70%)		1,951 (5.52%)	
50–59	43,074 (19.70%)	3,015 (7.38%)		2,562 (5.95%)	
60–69	47,524 (15.44%)	3,508 (7.92%)		3,255 (7.32%)	
≥70	52,658 (13.90%)	4,273 (8.72%)		5,445 (10.07%)	
Education level			<0.001		<0.001
<middle school	75,471 (19.46%)	6,785 (10.32%)		7,806 (11.05%)	
high school	77,094 (36.39%)	5,230 (7.26%)		4,828 (6.57%)	
≥college	76,514 (44.10%)	4,306 (5.72%)		3,424 (4.47%)	
Income level (monthly)			<0.001		<0.001
<3 million won	106,599 (34.69%)	9,749 (10.25%)		10,592 (10.35%)	
≥3 million won	122,614 (65.28%)	6,583 (5.54%)		5,478 (4.48%)	
Marital status			<0.001		<0.001
Married	151,425 (63.75%)	9,195 (6.21%)		8,492 (5.30%)	
Widowed	27,101 (7.10%)	2,917 (12.08%)		3,447 (12.62%)	
Divorced	11,060 (4.78%)	1,488 (14.32%)		1,528 (14.60%)	
Single	39,558 (24.35%)	2,727 (6.87%)		2,597 (6.34%)	
Self-rated health			<0.001		<0.001
Very good	14,649 (7.61%)	531 (3.58%)		415 (2.67%)	
Good	78,385 (37.81%)	3,104 (4.09%)		2,442 (3.20%)	
Fair	97,378 (42.38%)	6,522 (7.37%)		5,980 (6.39%)	
Poor	31,657 (10.30%)	4,348 (16.24%)		4,815 (16.57%)	
Very poor	7,138 (1.89%)	1,826 (29.74%)		2,417 (36.32%)	
Household composition			<0.001		<0.001
Living with others	190,413 (86.33%)	12,085 (6.48%)		11,379 (5.70%)	
Living alone	38,800 (13.65%)	4,247 (11.56%)		4,691 (11.67%)	
Residential area			0.001		
Rural area	100,033 (18.75%)	6,693 (6.76%)		7,272 (6.82%)	0.650
Urban area	129,180 (81.24%)	9,639 (7.27%)		8,798 (6.45%)	

Note: Values are presented as unweighted numbers with weighted percentages in parentheses. P-values were calculated using the Rao-Scott chi-square test.

### COVID-19-related life changes and mental health outcomes

Table 2 presents pandemic-related changes in participants’ lives and their association with mental health outcomes. During the pandemic, 5.58% of participants reported no change in their daily lives, while 1.53% reported complete disruption (score 100). Depression and suicidal thoughts were more prevalent among those reporting either no change or complete disruption compared to those reporting minor changes (scores 10–50).

**Table 2:** COVID-19-related life changes by mental health status.

	Total	Depression		Suicidal thoughts	
	n (weighted %)	n (weighted %)	P value	n (weighted %)	P value
Total	229,213 (100.00%)	16,332 (7.17%)		16,070 (6.52%)	
Changes in daily life			<0.001		<0.001
0 (unchanged)	17,363 (5.58%)	1,265 (7.60%)		1,526 (7.95%)	
10	11,983 (4.73%)	727 (6.25%)		769 (6.10%)	
20	26,033 (11.02%)	1,370 (5.22%)		1,409 (4.83%)	
30	33,199 (14.92%)	1,717 (5.03%)		1,636 (4.47%)	
40	29,173 (13.28%)	1,719 (5.89%)		1,631 (5.15%)	
50	56,303 (24.58%)	3,947 (6.86%)		3,847 (6.28%)	
60	15,709 (7.16%)	1,339 (8.66%)		1,302 (8.10%)	
70	20,352 (9.83%)	1,821 (8.96%)		1,742 (8.10%)	
80	9,289 (4.55%)	1,018 (11.11%)		900 (9.23%)	
90	5,721 (2.75%)	747 (14.10%)		691 (11.45%)	
100 (completely disrupted)	3,815 (1.53%)	638 (17.01%)		586 (15.60%)	
Changes in employment			<0.001		<0.001
Unchanged	128,472 (54.32%)	7,174 (5.48%)		6,763 (4.74%)	
Lost a job	7,190 (3.69%)	974 (14.18%)		915 (12.79%)	
Worsened working conditions	34,575 (16.69%)	2,848 (8.76%)		2,586 (7.65%)	
Improved working conditions	3,733 (1.97%)	260 (6.82%)		261 (7.06%)	
Not employed	51,934 (21.78%)	4,741 (8.88%)		5,265 (8.88%)	
Changes in household income			<0.001		<0.001
Decreased	80,666 (37.26%)	6,617 (8.33%)		74,578 (7.50%)	
Unchanged	142,666 (59.76%)	9,252 (6.41%)		133,129 (5.89%)	
Increased	5,774 (2.93%)	455 (7.99%)		5,350 (6.75%)	
Changes in physical activity			<0.001		<0.001
Increased	16,543 (8.25%)	1,230 (7.32%)		1,047 (5.91%)	
Unchanged	107,882 (43.48%)	6,040 (5.61%)		6,254 (5.34%)	
Decreased	91,474 (44.35%)	7,708 (8.41%)		7,285 (7.37%)	
No regular exercise	13,298 (3.93%)	1,352 (10.29%)		1,480 (11.08%)	
Changes in instant food consumption			<0.001		<0.001
Increased	33,397 (19.75%)	2,820 (8.32%)		2,528 (7.23%)	
Unchanged	112,552 (49.16%)	6,437 (5.82%)		6,857 (5.61%)	
Decreased	21,997 (9.44%)	1,916 (8.80%)		1,677 (6.97%)	
Non-consumer of instant foods	61,254 (21.62%)	5,157 (8.52%)		5,004 (7.72%)	
Changes in alcohol consumption			<0.001		<0.001
Increased	10,170 (5.42%)	1,100 (11.07%)		994 (9.81%)	
Unchanged	64,782 (29.99%)	3,687 (5.87%)		3,649 (5.38%)	
Decreased	56,264 (29.01%)	3,506 (6.35%)		3,209 (5.59%)	
Non-drinker	97,978 (35.57%)	8,034 (8.36%)		8,213 (7.73%)	
Changes in smoking amount			<0.001		<0.001
Increased	5,507 (2.85%)	786 (14.74%)		763 (13.74%)	
Unchanged	36,224 (17.64%)	2,154 (6.10%)		2,230 (5.86%)	
Decreased	10,866 (5.14%)	828 (7.81%)		804 (7.29%)	
Non-smoker	176,585 (74.36%)	12,559 (7.10%)		12,266 (6.34%)	

Note: Values are presented as unweighted numbers with weighted percentages in parentheses. P-values were calculated using the Rao-Scott chi-square test

Regarding employment status, 54.32% reported no changes, 3.69% lost jobs, 16.69% experienced worse working conditions, 1.97% reported improved conditions, and 21.78% were unemployed before the pandemic. For household income, 37.26% reported decreases, 59.76% reported no change, and 2.93% reported increases. Depression and suicidal thoughts were more prevalent among those reporting any change in employment or income compared to those reporting no changes.

Changes in health behaviors varied: physical activity increased in 8.25% and decreased in 44.35% of participants; instant food consumption increased in 19.75% and decreased in 9.44%; alcohol consumption increased in 5.42% and decreased in 29.99%; and for smoking, 2.85% reported increases, 5.14% reported decreases, while 74.36% were non-smokers. Depression and suicidal thoughts were more common among those reporting behavioral changes, particularly among those with increased alcohol consumption and smoking during the pandemic.

### Associations between pandemic-related changes and mental health

**Fig 1:**
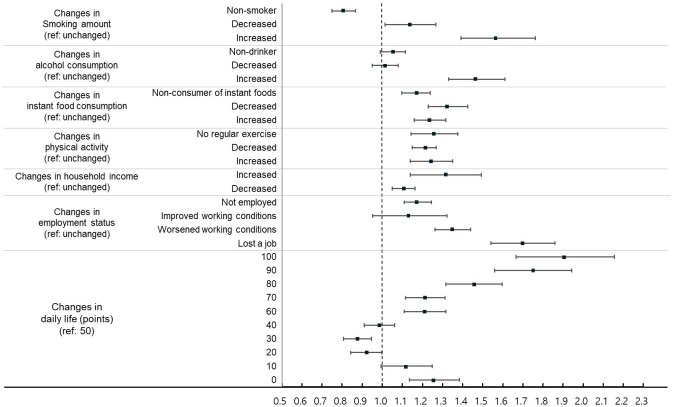
Adjusted odds ratios for depression by pandemic-related changes. Note: Adjusted odds ratios (aORs) with 95% confidence intervals (CIs) are presented adjusting for sex, age group, educational level, income level, marital status, residential area, self-reported health, and household composition.

**Fig 2:**
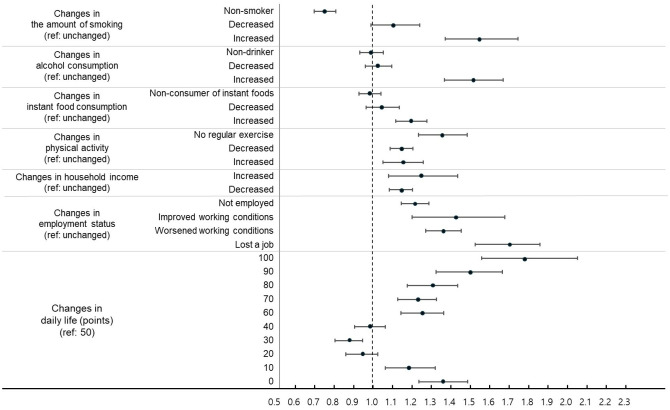
Adjusted odds ratios for suicidal thoughts by pandemic-related changes. Note: Adjusted odds ratios (aORs) with 95% confidence intervals (CIs) are presented adjusting for sex, age group, educational level, income level, marital status, residential area, self-reported health, and household composition.

After adjusting for demographic and socioeconomic factors (sex, age, education, income, marital status, residential area, self-rated health, and household composition), various types of pandemic-related changes showed significant associations with mental health outcomes (Figs 1 and 2, S1 Table).

Both minimal and substantial changes in daily life were associated with poor mental health. Complete disruption of daily life (100 points) showed the highest risk of depression (aOR 1.895, 95% CI 1.667–2.155, P < 0.001) and suicidal thoughts (aOR 1.788, 95% CI 1.558–2.052, P < 0.001) compared with the reference group (50 points). Participants reporting no change in daily life (0 points) also showed significantly higher rates of depression (aOR 1.253, 95% CI 1.135–1.384, P < 0.001) and suicidal thoughts (aOR 1.355, 95% CI 1.236–1.486, P < 0.001).

Changes in economic conditions, regardless of their direction, were associated with adverse mental health outcomes. Job loss showed the strongest association with depression (aOR 1.693, 95% CI 1.541–1.860, P < 0.001) and suicidal thoughts (aOR 1.684, 95% CI 1.525–1.859, P < 0.001). Even participants reporting improved working conditions showed increased risk of suicidal thoughts (aOR 1.419, 95% CI 1.200–1.677, P < 0.001). Similarly, increased household income was associated with higher risks of depression (aOR 1.304, 95% CI 1.139–1.493, P < 0.001) and suicidal thoughts (aOR 1.244, 95% CI 1.079–1.435, P = 0.003), as was decreased income (depression: aOR 1.105, 95% CI 1.049–1.164, P < 0.001; suicidal thoughts: aOR 1.141, 95% CI 1.083–1.203, P < 0.001).

Among health behavior changes, increased alcohol consumption (depression: aOR 1.465, 95% CI 1.331–1.611, P < 0.001; suicidal thoughts: aOR 1.511, 95% CI 1.368–1.669, P < 0.001) and increased smoking (depression: aOR 1.567, 95% CI 1.392–1.763, P < 0.001; suicidal thoughts: aOR 1.547, 95% CI 1.372–1.745, P < 0.001) showed the strongest associations with poor mental health. Even positive changes in health behaviors were associated with adverse mental health outcomes: increased physical activity (depression: aOR 1.241, 95% CI 1.140–1.351, P < 0.001; suicidal thoughts: aOR 1.150, 95% CI 1.051–1.258, P = 0.002), decreased instant food consumption (depression: aOR 1.324, 95% CI 1.229–1.426, P < 0.001), and reduced smoking (depression: aOR 1.133, 95% CI 1.014–1.266, P = 0.027) were associated with higher risks of mental health problems.

## Discussion

This study explored changes in daily life, economic activities, and health behaviors related to COVID-19 in South Korea and their associations with mental health outcomes. Our findings demonstrated that the relationship between pandemic-related changes and mental health was more complex than previously understood, revealing non-linear patterns and unexpected associations with both negative and positive life changes.

The prevalence of depression and suicidal thoughts was higher among individuals who reported either no effect or substantial changes compared with those who experienced minor changes due to the pandemic. This finding differs from previous studies that assumed a linear relationship where only negative pandemic-induced changes led to worse mental health outcomes [38, 49–53]. Our results suggest that the impact of the pandemic on mental health follows a more complex pattern, where both extremes of the change spectrum–no change and complete disruption–are associated with adverse mental health outcomes.

Most notably, our analysis revealed an unexpected association between positive pandemic-induced changes and mental health conditions. Increases in household income or improved working conditions were associated with higher rates of depression and suicidal thoughts. This paradoxical finding may be explained by examining the experiences of frontline workers in sectors such as healthcare, sanitation, law enforcement, and delivery services. While these workers may have reported increased income or improved working conditions due to pandemic-related demands, they also faced increased workloads, heightened stress, and potential burnout. This interpretation is supported by previous research showing that healthcare workers experiencing frequent suicidal thoughts during the pandemic reported exacerbation of pre-existing mental health issues, despite increased work opportunities [39].

Our findings align with the multiple stressor framework presented in previous studies [2, 3]. The pandemic created various sources of stress: direct health-related concerns, social distancing measures, economic uncertainty, and widespread misinformation. Even positive changes in economic conditions could be accompanied by other stressors, such as increased workload, social isolation, or reduced access to health-promoting activities. This complexity suggests that mental health interventions during crises should consider both the nature and magnitude of life changes.

Furthermore, individuals reporting no changes in daily life despite widespread social distancing measures [59] may represent a particularly vulnerable group. These individuals were likely to have been socially isolated or minimally socially active even before the pandemic, suggesting pre-existing vulnerability that could be exacerbated during crisis situations.

Our study addresses several limitations of previous research. Unlike previous studies that analyzed pandemic-related changes as continuous variables [38], our approach captured non-linear relationships between life changes and mental health outcomes. Additionally, we distinguished between individuals who maintained pre-existing behaviors and those who reported no changes during the pandemic, providing a more detailed understanding of the relationship between behavioral changes and mental health.

These findings have important implications for disaster response policies, particularly in the context of South Korea’s mental health interventions during the pandemic [42–44]. While existing policies focus on providing psychological support and counseling services, our results suggest the need for more targeted interventions that consider both negative and positive life changes. Mental health support should extend beyond those experiencing obvious hardships to include individuals reporting no impact or even positive changes.

This study has several limitations. First, this study used cross-sectional survey data, which necessitates caution when interpreting the findings as evidence for causal relationships. Despite this cross-sectional design, the research provided valuable insights into the relationship between COVID-19-related changes and mental health, as it directly surveyed participants about their pandemic-related experiences. Second, the face-to-face interview methodology may have led to underreporting of sensitive topics, such as depression or suicidal thoughts, particularly when interviews were conducted in the presence of other household members. Third, we did not stratify our analysis by job profile, which could have provided more detailed insights into the relationship between workload changes and mental health across different occupational groups.

Despite these limitations, our study has significant strengths. The KCHS provides a large, nationally representative sample with rigorous data collection methods. Moreover, our findings reveal complex patterns in the relationship between pandemic-related changes and mental health that previous studies have overlooked.

This study highlights the need for comprehensive evaluation of individuals’ life changes when developing mental health interventions during crises. Given that the COVID-19 pandemic has now ended, future research should examine mental health trajectories across three distinct periods: pre-pandemic, during the pandemic, and post-pandemic periods. Such longitudinal studies would help understand both the immediate and lasting effects of the pandemic on mental health. Additionally, research is needed to evaluate the effectiveness of various mental health interventions implemented during the pandemic. These evaluations would provide valuable insights for developing more effective response strategies for future public health crises, particularly considering both negative and positive socioeconomic changes that may occur during such events.

## Supporting information

S1 Table Adjusted odds ratios for depression and suicidal thoughts by pandemic-related changes. (DOCX)
