# Supplementary material for: Unexpected effects of pandemic-related changes on mental health: Evidence from a nationwide survey in South Korea
Source: PLoS One. 2025 Jan 13;20(1):e0317493. doi: 10.1371/journal.pone.0317493 (PMC11729936; doi:10.1371/journal.pone.0317493)
Supplement: S1 Table — (DOCX) [file pone.0317493.s001.docx]

|  | Depression | | Suicidal thought | |
| --- | --- | --- | --- | --- |
|  | aOR (95% CI) | *P* value | aOR (95% CI) | *P* value |
| Changes in daily life |  |  |  |  |
| 0 (unchanged) | 1.253 (1.135, 1.384) | <0.001 | 1.355 (1.236, 1.486) | <0.001 |
| 10 | 1.114 (0.995, 1.248) | 0.062 | 1.184 (1.062, 1.320) | 0.002 |
| 20 | 0.918 (0.843, 0.999) | 0.048 | 0.938 (0.860, 1.023) | 0.152 |
| 30 | 0.874 (0.808, 0.946) | 0.001 | 0.872 (0.804, 0.946) | 0.001 |
| 40 | 0.983 (0.911, 1.061) | 0.665 | 0.980 (0.904, 1.062) | 0.640 |
| 50 | 1.000 (Reference) | - | 1.000 (Reference) | - |
| 60 | 1.209 (1.110, 1.317) | <0.001 | 1.248 (1.143, 1.363) | <0.001 |
| 70 | 1.210 (1.116, 1.313) | <0.001 | 1.223 (1.127, 1.327) | <0.001 |
| 80 | 1.450 (1.316, 1.597) | <0.001 | 1.298 (1.175, 1.435) | <0.001 |
| 90 | 1.740 (1.559, 1.943) | <0.001 | 1.484 (1.323, 1.665) | <0.001 |
| 100 (completely disrupted) | 1.895 (1.667, 2.155) | <0.001 | 1.788 (1.558, 2.052) | <0.001 |
| Changes in employment |  |  |  |  |
| Unchanged | 1.000 (Reference) |  | 1.000 (Reference) | - |
| Lost a job | 1.693 (1.541, 1.860) | <0.001 | 1.684 (1.525, 1.859) | <0.001 |
| Worsened working conditions | 1.347 (1.262, 1.439) | <0.001 | 1.358 (1.270, 1.453) | <0.001 |
| Improved working conditions | 1.122 (0.952, 1.323) | 0.17 | 1.419 (1.200, 1.677) | <0.001 |
| Not employed | 1.175 (1.110, 1.244) | <0.001 | 1.214 (1.145, 1.286) | <0.001 |
| Changes in household income |  |  |  |  |
| Decreased | 1.105 (1.049, 1.164) | <0.001 | 1.141 (1.083, 1.203) | <0.001 |
| Unchanged | 1.000 (Reference) | - | 1.000 (Reference) | - |
| Increased | 1.304 (1.139, 1.493) | <0.001 | 1.244 (1.079, 1.435) | 0.003 |
| Changes in physical activity |  |  |  |  |
| Increased | 1.241 (1.140, 1.351) | <0.001 | 1.150 (1.051, 1.258) | 0.002 |
| Unchanged | 1.000 (Reference) | - | 1.000 (Reference) | - |
| Decreased | 1.207 (1.149, 1.268) | <0.001 | 1.145 (1.087, 1.205) | <0.001 |
| No regular exercise | 1.255 (1.144, 1.377) | <0.001 | 1.354 (1.234, 1.485) | <0.001 |
| Changes in instant food consumption |  |  |  |  |
| Increased | 1.235 (1.160, 1.316) | <.001 | 1.193 (1.116, 1.276) | <0.001 |
| Unchanged | 1.000 (Reference) | - | 1.000 (Reference) | - |
| Decreased | 1.324 (1.229, 1.426) | <0.001 | 1.046 (0.965, 1.135) | 0.301 |
| Non-consumer of instant foods | 1.166 (1.098, 1.238) | <0.001 | 0.983 (0.928, 1.041) | 0.559 |
| Changes in alcohol consumption |  |  |  |  |
| Increased | 1.465 (1.331, 1.611) | <0.001 | 1.511 (1.368, 1.669) | <0.001 |
| Unchanged | 1.000 (Reference) | - | 1.000 (Reference) | - |
| Decreased | 1.012 (0.950, 1.079) | 0.710 | 1.026 (0.961, 1.096) | 0.430 |
| Non-drinker | 1.051 (0.990, 1.116) | 0.105 | 0.991 (0.932, 1.053) | 0.746 |
| Changes in smoking amount |  |  |  |  |
| Increased | 1.567 (1.392, 1.763) | <0.001 | 1.547 (1.372, 1.745) | <0.001 |
| Unchanged | 1.000 (Reference) | - | 1.000 (Reference) | - |
| Decreased | 1.133 (1.014, 1.266) | 0.027 | 1.106 (0.988, 1.239) | 0.081 |
| Non-smoker | 0.806 (0.749, 0.867) | <0.001 | 0.751 (0.697, 0.809) | <0.001 |
| -2 Log Likelihood | 20,058,585 |  | 18,145,096 | <0.001 |

Supplementary Table 1. Adjusted odds ratios for depression and suicidal thoughts by pandemic-related changes

Note: aOR, adjusted odds ratio; CI, confidence interval. Adjusted for sex, age group, educational level, income level, marital status, residential area, Self-rated health, and household composition.
